# Mapping right ventricular myocardial mechanics using 3D cine DENSE cardiovascular magnetic resonance

**DOI:** 10.1186/1532-429X-14-4

**Published:** 2012-01-11

**Authors:** Daniel A Auger, Xiaodong Zhong, Frederick H Epstein, Bruce S Spottiswoode

**Affiliations:** 1 MRC/UCT Medical Imaging Research Unit, Department of Human Biology, University of Cape Town, Cape Town, South Africa; 2 MR R&D Collaborations, Siemens Healthcare, Atlanta, GA, USA; 3 Departments of Radiology and Biomedical Engineering, University of Virginia, Charlottesville VA, USA; 4 Division of Radiology, University of Stellenbosch, Cape Town, South Africa

**Keywords:** displacement encoding, DENSE, right ventricle, cardiac function, strain

## Abstract

**Background:**

The mechanics of the right ventricle (RV) are not well understood as studies of the RV have been limited. This is, in part, due to the RV's thin wall, asymmetric geometry and irregular motion. However, the RV plays an important role in cardiovascular function. This study aims to describe the complex mechanics of the healthy RV using three dimensional (3D) cine displacement encoding with stimulated echoes (DENSE) cardiovascular magnetic resonance (CMR).

**Methods:**

Whole heart 3D cine DENSE data were acquired from five healthy volunteers. Tailored post-processing algorithms for RV mid-wall tissue tracking and strain estimation are presented. A method for sub-dividing the RV into four regions according to anatomical land marks is proposed, and the temporal evolution of strain was assessed in these regions.

**Results:**

The 3D cine DENSE tissue tracking methods successfully capture the motion and deformation of the RV at a high spatial resolution in all volunteers. The regional Lagrangian peak surface strain and time to peak values correspond with previous studies using myocardial tagging, DENSE and strain encoded CMR. The inflow region consistently displays lower peak strains than the apical and outflow regions, and the time to peak strains suggest RV mechanical activation in the following order: inflow, outflow, mid, then apex.

**Conclusions:**

Model-free techniques have been developed to study the myocardial mechanics of the RV at a high spatial resolution using 3D cine DENSE CMR. The consistency of the regional RV strain patterns across healthy subjects is encouraging and the techniques may have clinical utility in assessing disrupted RV mechanics in the diseased heart.

## Background

Right ventricular (RV) function may be impaired in a number of heart conditions, including myocardial infarction, congenital heart disease and cardiomyopathy [1]. The function of the RV may also be affected in diseases of the left ventricle (LV) where it is difficult to ignore the complex nature of ventricular interaction [2,3]. In the past, the importance of the LV in cardiac research has overshadowed the study of the RV. This neglect is, in part, because the RV is difficult to image. The wall of the RV myocardium is thin (2-5 mm) when compared to that of the LV (7-11 mm) [2]. Furthermore, the RV has a complex geometry, eccentric motion [1,4] and it is heavily trabeculated, thus it does not offer the clearly defined endocardial margins typically seen in the LV.

Various cardiovascular magnetic resonance (CMR) studies have assessed properties such as strain, motion and volumes of the RV. The CMR techniques used include turbo gradient echo and steady state free precession (SSFP) [5], myocardial tagging [4,6,7], phase contrast velocity encoding [8], strain encoded (SENC) CMR [9] and 2D multi-slice displacement encoding with stimulated echoes (DENSE) [10].

The standard cardiac imaging planes are based on the relatively uncomplicated geometry of the LV. However, no standardized localization exists for the RV. Two dimensional strain estimates of the LV are reliable in short axis views because the through plane motion is relatively uniform [11]. This does not apply to the RV because of its complex shape and motion, so 2D imaging may be insufficient to accurately assess strain in the RV [4]. To date, no detailed studies of the RV have been presented using DENSE CMR.

DENSE is a quantitative CMR technique used for measuring myocardial displacement and strain. DENSE encodes tissue displacement directly into the image phase (typically with reference to end diastole), thus allowing for the extraction of motion data at a pixel resolution [12,13]. A recently developed free breathing 3D cine DENSE sequence [14] is well suited for quantifying the complex behavior of the RV. A high signal to noise ratio (SNR), which is necessary for imaging the thin RV walls, is achieved using a spiral k-space trajectory and three point phase cycling. Furthermore, DENSE is inherently a black-blood technique, which together with fat suppression, provides a more accurate delineation of the RV walls.

The purpose of this study was to develop tailored processing techniques for assessing detailed 3D RV motion and surface strain using 3D cine DENSE, and to quantify these parameters for the healthy human heart.

## Methods

### Imaging protocol

Whole heart 3D cine DENSE data were acquired from five healthy male volunteers (age range 21 - 45) on a 1.5T MRI scanner (Siemens MAGNETOM Avanto, Erlangen, Germany) using a two-channel anterior body array coil and an eight-channel spine array coil.

The entire heart was imaged with the imaging volume aligned along the heart's long axis and at a 2.8 × 2.8 × 5 mm^3 ^spatial resolution and 32 ms temporal resolution. Displacement was encoded in three orthogonal directions and a spiral k-space trajectory was used with 6 interleaves per 3D partition. Spiral aliasing artifacts existed on the edge of images, but not on the heart, which was placed at the center of the field of view. Images were acquired during a 20.5 ± 5.7 min scan time with prospective ECG gating, diaphragmatic navigator respiratory gating, and no multichannel parallel imaging. Other imaging parameters include: field of view (FOV) = 360 × 360 × 140 mm^3 ^(in-plane dimensions defined by the diameter of the spiral), displacement encoding frequency k_e _= 0.06 cycles/mm, ramped flip angle up to 20 degrees, TR = 16 ms, TE = 1.3 ms, cardiac phases = 22 and number of acquired 3D partitions = 14 (zero-padded to 28 during image reconstruction). After the Fourier Transform in the partition direction, three partitions at each end of the volume were discarded to avoid aliasing. The resulting 3D image matrix size was 128 × 128 × 22. The number of partitions chosen per subject in order to include the RV was approximately 14, which corresponds to an overall stack thickness of 70 mm. All imaging was conducted after informed consent and in accordance with protocols approved by the University of Virginia Institutional Review Board.

### Post processing

A number of tailored post processing steps were implemented to analyze the RV myocardial DENSE data. All software development was performed using MATLAB (The Mathworks, Natick, MA).

### Contouring, phase unwrapping and tissue tracking

Both the RV and LV myocardium was demarcated from surrounding structures by manually drawing each epicardial and endocardial contour on the reconstructed short-axis DENSE magnitude images.

**Figure 1:**
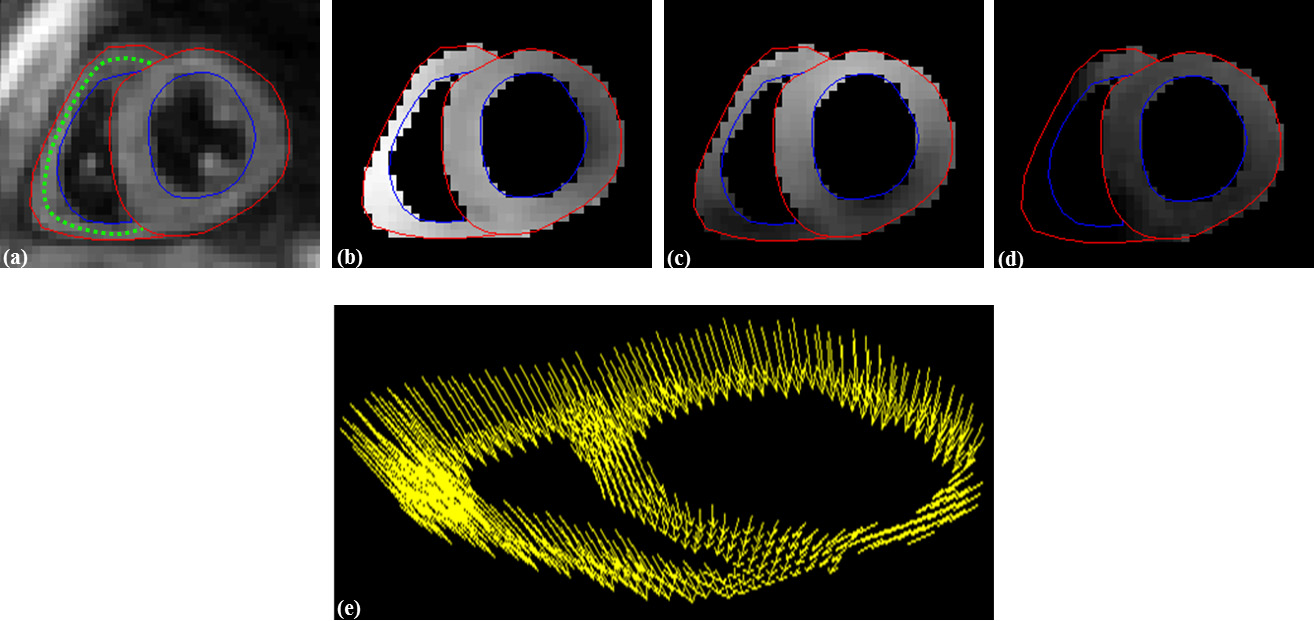
**A single short axis slice of the 3D cine DENSE data at mid systole**. (a) DENSE magnitude image illustrating the myocardial mid-line for the RV (green dotted line). (b, c, d) DENSE unwrapped phase images encoded for motion in the x, y and z directions, respectively. (e) Corresponding 3D DENSE displacement field. Red lines correspond to epicardial surfaces and blue lines correspond to endocardial surfaces.

Spatio-temporal phase unwrapping [15] was performed within the contoured area to remove phase aliasing, and the phase data from the three encoding directions were combined to create 3D Eulerian displacement fields. Figure 1 illustrates contoured cine DENSE magnitude and phase-unwrapped images and the corresponding 3D displacement fields at end systole for a single short-axis slice.

There are typically too few transmural pixels spanning the RV to acquire a full 3D strain tensor, so 2D strain at the RV mid-wall surface was calculated. The mid-line between epicardial and endocardial contours was used to produce tissue tracking seed points, which were spaced at pixel-distance intervals on the first cardiac phase. The mid-line contours were spatially smoothed within and across partitions using 4^th ^order polynomial fitting to ensure a continuous RV surface. An example of the RV mid-line points is shown in green in Figure 1(a).

A 2D tissue tracking algorithm involving interpolation of the displacement fields [15] was directly extended to 3D for this application. The position of each mid-line point along its motion trajectory throughout the cardiac cycle was estimated using 3D distance weighted linear interpolation. The full 3D mid-line motion trajectories were calculated by subtracting the interpolated vectors of successive frames from one another. Prospective gating only allowed about 90 percent of the cardiac cycle to be imaged, therefore, temporal fitting was done for each ordinate direction of each of the trajectories using a 10^th ^order polynomial, as 5^th ^order Fourier basis functions have been shown to sufficiently describe cardiac motion [16].

### Lagrangian surface strain

**Figure 2:**
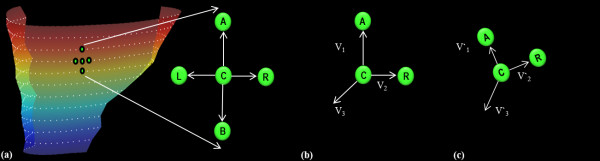
**2D Lagrangian surface strain estimation**. (a) RV surface image with superimposed mid-line points (white dots) representing the partitions. All possible vector configurations are shown for a chosen point (C) using mid-line points (A)bove, (B)elow, to the (L)eft and to the (R)ight. (b, c) Pre- and post-deformation, respectively, for a single vector configuration. Strain for the chosen point C is calculated using the configuration of points above and to the right (ACR). **V_1 _**and **V_2 _**represent the co-planar vectors formed. **V_3 _**represents the orthogonal unit vector calculated from the cross product of **V_1 _**and **V_2_**. **V'_1 _**and **V'_2 _**represent the corresponding post deformation co-planar vectors, and **V'_3 _**is the cross product of **V'_1 _**and **V'_2_**.

Lagrangian strain was calculated directly from the 3D motion trajectories and oriented tangential to the RV mid-line surface. Considering an arbitrary point on the mid-wall, the deformation gradient tensor **F **was calculated using two to four of the nearest neighboring mid-line points as follows

(1) $$F=\left[V_{1}^{\prime }V_{2}^{\prime }V_{3}^{\prime }\right]/)\left[V_{1}V_{2}V_{3}\right]$$

where V_1_, V_2 _and V_3 _represent the pre-deformation vectors which are perpendicular to each other in 3D space as shown in Figure 2 and form the columns of one matrix. V'_1_, V'_2 _and V'_3 _represent the corresponding post-deformation vectors and form the columns of another matrix, and the "/" operator is a matrix right division.

In practice, depending on the position of the mid-line point on the RV wall, each motion trajectory could have adjacent points to the right and/or left, and on partitions above and/or below. The deformation gradient tensor is therefore calculated for a minimum of two and a maximum of four co-planar vector configurations for each mid-line point. An example of four co-planar vector configurations is illustrated in Figure 2a, and an example of two co-planar vector configurations pre- and post-deformation is illustrated in Figure 2b and 2c, respectively. Orthogonal unit vectors (e.g. V3 and V'3) were created perpendicular to the RV surface using cross products of each set of co-planar vectors (e.g. V1 and V2, V'1 and V'2). Three dimensional Lagrangian strain was then calculated for each deformation gradient configuration as follows

(2) $$\text{E}=\frac{1}{2}\left({\text{F}}^{\text{T}}\text{F}-\text{I}\right)$$

where T denotes the transpose operation, and I represent the identity matrix. Note that this construction of a 3D tensor using a unit normal vector assumes that the cardiac tissue is non-compressible and has been implemented here to simplify the strain calculations. The 3D strain tensor was decomposed into its corresponding eigenvalues (strain) and eigenvectors (direction of deformation). The eigenvalue corresponding to the orthogonal unit vector (E_1_) is aligned with the direction normal to the surface (radial) and is zero because of the aforementioned unit normal vector assumption. The remaining two eigenvalues (E_2 _and E_3_) provide an estimate of the 2D tangential surface strain. The eigenvalues E_2 _and E_3 _are of similar magnitude, and there are regional differences in the myofiber (and eigenvector) orientation across the RV surface, so we were unable to discern between E_2 _and E_3_. The results of the surface strain analysis are thus presented as a mean Lagrangian 2D principal strain, which has been averaged for the various co-planar vector configurations.

It has been shown that tangential strain in the circumferential and longitudinal directions are good indicators of LV dyssynchrony [17]. In order to infer details about the strain directions in the RV, one dimensional (1D) strains were calculated in the longitudinal and circumferential directions in the same manner as usually done for the LV, and relative to the orientation of the LV. Each 1D strain was calculated from the motion trajectories by only considering points above and below (longitudinal) or to the left and right (circumferential) of each mid-line point. This is also illustrated in Figure 2.

### Anatomical sub-divisions for strain-time analyses

Unlike the LV, there is currently no standard method for dividing the RV into anatomical sub-regions. Previous studies of the RV using myocardial tagging have adopted different conventions for sub-dividing the RV. Klein et al. divides each RV basal, mid and apical short axis slice into three regions: superior, mid and inferior [18]. Haber et al. [4], divided the RV into four regions according to anatomical landmarks. The parietal and septal bands were used to demarcate the outflow region, while the free wall was identified based on the normalized height of the septum in a long axis view [4]. Fayad et al. divides the RV using the supraventricular crest (SC), moderator band, papillary muscles and the tricuspid valve as landmarks, creating four distinct regions: the inflow, outflow, mid and apical regions [19].

**Figure 3:**
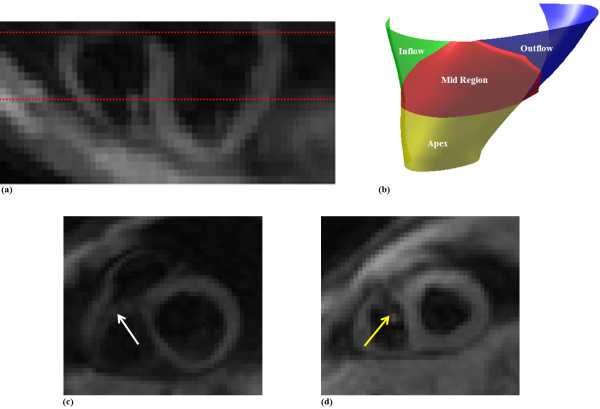
**Right ventricular regional divisions**. (a) Long axis view showing the level of the SC (top red dotted line) and moderator band (bottom red dotted line). (b) RV surface showing the four anatomical regions. (c, d) Short axis view corresponding to the partitions at the level of the SC and moderator band, respectively. The white and yellow arrows show the SC and moderator band, respectively.

We propose dividing the RV surface in a similar manner to Fayad et al. These regions are illustrated in Figure 3. The divisions were defined by navigating through the 3D DENSE magnitude-reconstructed images and identifying three landmark coordinates as follows:

Coordinate 1: The SC is an accentuation of the muscular wall demarcating the inflow and outflow tracts within the RV. Navigate from apex to base along the short axis magnitude images until the SC appears in the image. The first coordinate is marked on the RV free wall at the level of and adjacent to the SC.

Coordinates 2 and 3: The moderator band is a muscular band connecting the interventricular septum to the anterior papillary muscle of the tricuspid valve. Navigate from base to apex along the short axis magnitude images and define the slice where the moderator band becomes evident as a segment of myocardium crossing the RV cavity. The second and third coordinates are defined as the anterior and inferior RV-LV insertion points at this level, respectively.

The apical RV region is defined as the myocardium lying apical to the slice identified by the moderator band. The inflow, mid and outflow RV regions are defined using two planes: (i) a plane defined by coordinate 1, coordinate 2 and a point displaced normal to the RV mid-line surface in this region, and (ii) a plane defined by coordinate 1, coordinate 3 and a point displaced normal to the RV mid-line surface in this region. The strain values (2D and 1D Lagrangian) were averaged in each of the 4 segments and assessed through the duration of the cardiac cycle for all 5 volunteers.

## Results

**Figure 4:**
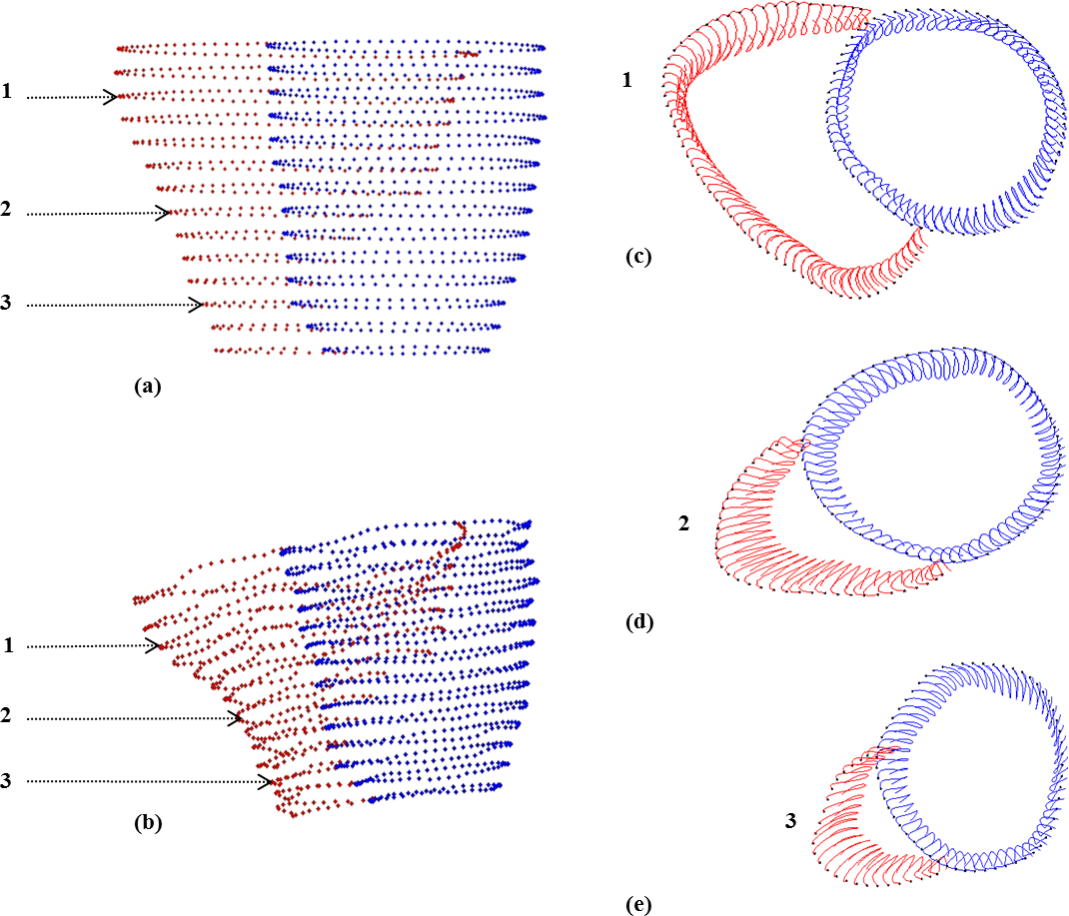
**3D mid-line motion trajectories**. (a, b) show the RV (red) and LV (blue) positions during end-diastole and end-systole, respectively. (c, d and e) represent the motion trajectories for the respective slices 1,2 and 3.

The frame-to-frame 3D mid-line motion trajectories for both the LV and RV are illustrated in Figure 4. The red and blue colors represent the positions along the motion trajectories for the RV and LV, respectively. The evolution of displacement during the cardiac cycle can be appreciated in detail, and the large range of motion of the RV compared to the LV is evident.

**Figure 5:**
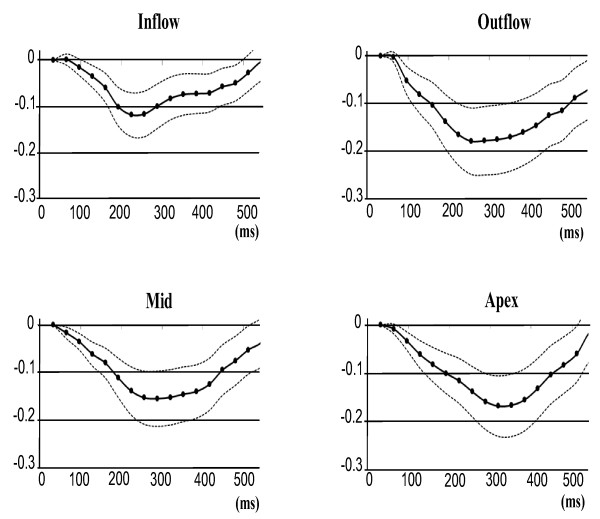
**Mean principal 2D Lagrangian regional RV surface strain-time curves for 5 normal volunteers**.

**Figure 6:**
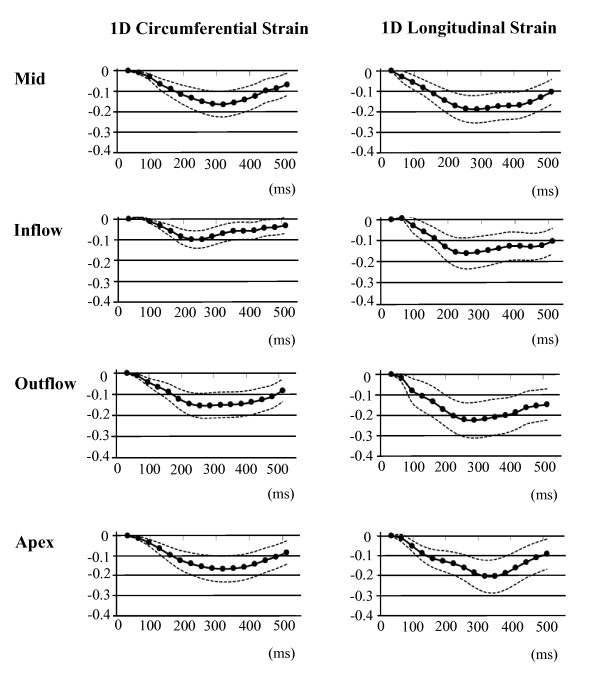
**1D Lagrangian regional RV surface strain-time curves for 5 normal volunteers**.

The regional mean RV midwall strain-time curves for the 5 volunteers are plotted in Figures 5 and 6. Figure 5 shows the mean of the principal strains E_2 _and E_3_, while the 1D tangential circumferential and longitudinal strains are shown in Figure 6. The data are represented as mean ± one standard deviation. A summary of the maximum strain and the time to peak strain for each RV anatomical region is given in Table 1.

**Table 1:** Right ventricular regional Lagrangian strain and time to peak strain

Strain quantification method and anatomical region	Maximum strain	Time to peak (ms)
**1D Circumferential**		
Inflow	-0.10 ± 0.04	224
Outflow	-0.15 ± 0.05	256
Apex	-0.17 ± 0.06	320
Mid Region	-0.16 ± 0.06	320
**1D Longitudinal**		
Inflow	-0.16 ± 0.06	256
Outflow	-0.22 ± 0.08	288
Apex	-0.20 ± 0.07	352
Mid Region	-0.18 ± 0.06	288
**Mean principal strain**		
Inflow	-0.12 ± 0.05	224
Outflow	-0.18 ± 0.07	256
Apex	-0.17 ± 0.06	320
Mid Region	-0.16 ± 0.05	288

All strain values are consistently negative, indicating muscle shortening or contraction, and the longitudinal shortening is greater for all regions when compared to circumferential shortening.

The inflow region has the lowest peak strain value of all the regions, while the outflow region generally reaches the highest peak strain. However, Figure 6 illustrates that during tangential shortening in the circumferential direction, the apical region tends to have a slightly greater strain than the outflow region. Good consistency between the patterns of strain evolution is shown for the various strain estimates in Figures 5 and 6. The four regions each arrive at a maximum strain at slightly different time points in the cardiac cycle. The time sequence of regional myocardial shortening, as confirmed by all strain estimates is: inflow, outflow, mid then apical.

**Figure 7:**
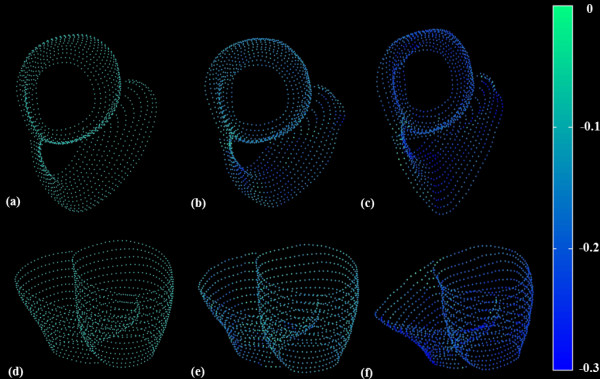
**Tissue tracking and mid-wall strain**. LV and RV mid-line images representing mean principal 2D Lagrangian surface strain in two views. (a, d) End-diastole, (b, e) mid-systole and (c, f) end-systole. The dots represent the motion trajectory positions, while color represents mean principal strain.

Figure 7 demonstrates 3D cine DENSE bi-ventricular midwall tissue tracking in two views for one volunteer at end-diastole, mid-systole and end-systole, with the tracked points colour coded according the mean principal strain. The additional files 1 and 2 show these data for all cardiac phases in plane and oblique views, respectively.

## Discussion

This paper introduces post processing algorithms developed for 3D tissue tracking and strain analysis of the RV from 3D cine DENSE data. Unlike previous studies of 3D RV motion using tagging [4,7], no models were used to describe the underlying data. Furthermore, no spatial smoothing was applied to the data, and the only form of filtering was the temporal polynomial fitting applied to the mid-line motion trajectories. This temporal fitting provides a more realistic trajectory behavior and allows for motion to be estimated at any temporal resolution.

Both the peak strain and the time to peak strain were found to vary across the different RV regions. The inflow region consistently demonstrated lower strains and early strain peak times, while the apex consistently showed larger strain values and later peak times. Some variation does exist in the outflow region when comparing the different strain calculation types.

As there is no current standard for dividing the RV, and multiple approaches for calculating strain have been adopted, it is difficult to make a direct comparison with results from previous studies. In general, however, the magnitude trends and timing parameters of the strain measurements from this study compare favorably with previous RV studies using myocardial tagging, SENC and DENSE. From our study the peak mean principal strains values in the apical, mid and outflow regions are -0.17, -0.16 and -0.18, respectively. These values are similar to the corresponding values of -0.17, -0.19 and -0.22 reported in a tagging study by Haber et al. [4].

In the SENC study by Hamdan et al. [9], they show longitudinal strains to be largest at the base, with a lower strain at the apex, and the lowest strain in the mid region. For circumferential strain they show the largest strain at the apex, with a lower strain in the mid and basal regions. These regional strain comparisons show excellent agreement with those presented in Table 1. In our results, during longitudinal contraction, the largest strain is found in the outflow region (basal), followed by the apical and mid regions, while circumferential strain in the apical region is larger, followed by the mid and outflow (basal) regions.

The 1D peak longitudinal strains in Table 1 are all larger than the corresponding 1D peak circumferential and mean principal strains, a result confirmed in tagging studies by both Haber et al [4] and Fayad et al. [19]. Fayad et al., who used a very similar method to ours for sub-dividing the RV, found regional peak circumferential strains to range from largest to smallest in the following order: apical, mid, inflow and outflow. For peak longitudinal strains the corresponding order was: outflow, inflow, apical and mid regions. The peak strain values in Table 1 also correlate well with the segmental shortening presented by Fayad et al., except for the inflow region where our values are consistently lower.

Wen et al. [10] present circumferential strain results from 2D DENSE data as an average of the entire RV free wall, in three slices: basal, mid and apical. Here, the strain is derived from the lengthening of the RV contour. They also found decreasing peak circumferential strain from apex to base, but the results plotted in [10] have a consistently larger magnitude than those presented here.

Limitations of our study include a lengthy scan time, the need for manual contouring, and the difficulty to discern between the minimum and intermediate principal strains. A reduction in the scan time could be accomplished by incorporating parallel imaging and/or outer volume suppression. The time taken to contour the images may be considerably reduced with the extension of previous work on motion guided segmentation for 2D cine DENSE images [20]. Even with the use of the eigenvectors, discerning between the two principal strains was not possible. A geometric or finite element model of the RV could solve this problem, whereby the equivalent of an RLC coordinate system for the LV is defined according to the RV geometry.

The 2D surface strain estimates are expected to have a lower accuracy in the through-plane direction because the through-plane image resolution is lower than the in-plane resolution. This should lead to a less accurate representation of strain in the longitudinal direction compared to the circumferential direction, but this is not evident in the standard deviation curves in Figure 6.

An inherent limitation of DENSE is the reduction in SNR associated with the stimulated echo. The SNR was typically sufficient to clearly identify the RV because of the short echo times associated with the spiral readout. However, signal loss due to through-plane dephasing is particularly pronounced in the RV because of its large through-plane strain [15]. This reduction in SNR was evident in a few instances, and these data had to be excluded from the analyses. For future work on the RV, this through-plane dephasing could be minimized by lowering the displacement encoding frequency, but at the expense of displacement sensitivity.

## Conclusions

Three dimensional cine DENSE CMR is a promising technique for assessing RV mechanics. The methods presented here enable the motion and deformation of the entire RV to be captured at a pixel resolution. The RV mechanics measured by 3D cine DENSE is comparable to that of previous studies using tagging, SENC and 2D DENSE.

We further describe a method for dividing RV into regions according to anatomical land marks. The differing temporal strain evolution in the proposed RV anatomical sub-divisions indicates that these regions may also be functionally distinct. Future work will involve applying these techniques to studying variations in regional RV function in cardiac disease.

## Competing interests

XZ is an employee of Siemens Medical Solutions USA, Inc. FHE received research funding from Siemens Medical Solutions.

## Authors' contributions

XZ and FHE developed and implemented the 3D spiral cine DENSE sequence and acquired the data. BSS and XZ participated in the development of post-processing software. DAA implemented all RV-specific post processing techniques. DAA and BSS drafted the manuscript and figures. All authors read and approved the final manuscript.

## Supplementary Material

Additional file 1 **Three dimensional mid-line motion trajectories in a plane view of the LV and RV seeded from the mid-line points, for each partition/slice**. Motion trajectories begin at end diastole. The dots represent the motion trajectory positions, while the color represents mean principal 2D Lagrangian surface strain. Click here for file

Additional file 2 **Three dimensional mid-line motion trajectories in an oblique view of the LV and RV seeded from the mid-line points, for each partition/slice**. Motion trajectories begin at end diastole. The dots represent the motion trajectory positions, while the color represents mean principal 2D Lagrangian surface strain. Click here for file
